# Comparative Analysis of *Viperidae* Venoms Antibacterial Profile: a Short Communication for Proteomics

**DOI:** 10.1093/ecam/nen052

**Published:** 2011-06-05

**Authors:** Bruno L. Ferreira, Dilvani O. Santos, André Luis dos Santos, Carlos R. Rodrigues, Cícero C. de Freitas, Lúcio M. Cabral, Helena C. Castro

**Affiliations:** ^1^Departamento de Biologia Celular e Molecular, Laboratório de Antibióticos, Bioquímica e Modelagem Molecular (LABioMol), Instituto de Biologia, CEG, Universidade Federal Fluminense, CEP 24001–970, Niterói, Brazil; ^2^Cursos de Pós-graduação em Neuroimunologia - IB, e Patologia - HUAP, Universidade Federal Fluminense, CEP 24001-970, Niterói, RJ, Brazil; ^3^Laboratório de Modelagem Molecular e QSAR (ModMolQSAR), Faculdade de Farmácia, Universidade Federal do Rio de Janeiro, CEP 21941-590, Rio de Janeiro, RJ, Brazil

## Abstract

Bacterial infections involving multidrug-resistant strains are one of the ten leading causes of death and an important health problem in need for new antibacterial sources and agents. Herein, we tested and compared four snake venoms (*Agkistrodon rhodostoma, Bothrops jararaca, B. atrox and Lachesis muta*) against 10 Gram-positive and Gram-negative drug-resistant clinical bacteria strains to identify them as new sources of potential antibacterial molecules. Our data revealed that, as efficient as some antibiotics currently on the market (minimal inhibitory concentration (MIC) = 1–32 *μ*g mL^−1^), *A. rhodostoma* and *B. atrox* venoms were active against *Staphylococcus epidermidis* and *Enterococcus faecalis* (MIC = 4.5 *μ*g mL^−1^), while *B. jararaca* inhibited *S. aureus* growth (MIC = 13 *μ*g ml^−1^). As genomic and proteomic technologies are improving and developing rapidly, our results suggested that *A. rhodostoma, B. atrox* and *B. jararaca* venoms and glands are feasible sources for searching antimicrobial prototypes for future design new antibiotics against drug-resistant clinical bacteria. They also point to an additional perspective to fully identify the pharmacological potential of these venoms by using different techniques.

## 1. Introduction

Bacterial infections are among the 10 leading causes of death worldwide according to the World Health Organization [1, 2]. The presence and current emergence of the kept multiple-resistant strains make the risk of these infections become more threatening as the treatment becomes unreachable. In fact, bacterial resistance has been the major factor responsible for increasing morbidity, mortality and health care costs of bacterial infections [1–5]. Therefore, new antimicrobials or antibacterial prototypes are continuously necessary for drug design and development for treatment of infections involving multidrug-resistant microorganisms [1, 2, 6, 7].

Snake venoms are a complex mixture of proteins and peptides that display potential biological activities and may lead to the production of new drugs of potential therapeutic value [8, 9]. A good example is the bradykinin-potentiating peptides (BPPs), which are naturally occurring inhibitors of the somatic angiotensin-converting enzyme (ACE) found in *Bothrops jararaca* venom [10]. The chemical and pharmacological properties of these peptides were essential for the development of captopril, the first active site directed inhibitor of ACE, currently used to treat human hypertension [11].

Lately, several naturally occurring peptides presenting antimicrobial activity have been described in the literature. However, *Viperidae* snake venoms that are an enormous source of peptides have not been fully explored for searching such biological activity [5, 12]. Therefore, in this study, we tested the antibiotic profile of four snake venoms from three different genera of *Viperidae* family (*Agkistrodon rhodostoma, Bothrops atrox, B. jararaca and Lachesis muta*) against 10 drug-resistant Gram-positive and Gram-negative bacteria clinical strains. Based on captopril history, our purpose is to identify some of these venoms as potential source for finding new and effective antibacterial prototypes.

## 2. Materials and Methods

### 2.1. Materials

Each venom was purchased from Sigma (St Louis, MO, USA) and one *L. muta* venom sample was also obtained from each Brazilian governmental sources—Butantan Institute, SP, Ezequiel Dias Institute, SP and Vital Brazil Institute, RJ, Brazil. The 10 Gram-positive (*Enterococcus faecalis, Staphylococcus epidermidis* and *S. aureus*) and Gram-negative (*Escherichia coli, Serratia marcenses, Proteus mirabilis, Pseudomonas aeruginosa, Enterobacter cloacae, Acinetobacter calcoaceticus and Klebsiella pneumoniae*) drug-resistant clinical bacteria were isolated from patients of the Hospital Antônio Pedro from Fluminense Federal University. All other reagents were from Sigma. After isolation, the bacterial strains were kept frozen in 10% milk-sterilized solution containing 10% glycerin.

### 2.2. Methods

#### 2.2.1. Sensibility Test

The test and MIC were performed according to the National Committee for Clinical Laboratory Standards (NCCLS), in Müeller-Hinton medium as described elsewhere [13]. Briefly, the strains were grown at 37°C in Müeller-Hinton medium. Then, 1 *μ*L of the snake venom solutions prepared with sterilized distilled and deionized water (20 mg mL^−1^) was placed in Whatman disks (5 mm diameter). The disks were put on an exponentially growing plated culture with appropriate dilution to 1.0 × 10^7^ colony forming unit (CFU mL^−1^), which were then incubated for 24 h at 37°C. The inoculums used in growth method were those where turbidity was equal to 0.5 McFarland Standard. The results were verified by measuring the zones surrounding the disk. Ciprofloxacin and vancomycin were used as positive controls and the halo >15 mm was considered the minimum value for positive antimicrobial activity as it generally leads to a MIC near to that observed for the newest antimicrobials current present on the market (MIC = 1−40 *μ*g  mL^−1^). Vancomycin and ciprofloxacin presented halo 15 ± 2 and 23 ± 2 mm, respectively in the strains tested herein (*P* < .005).

#### 2.2.2. MIC Assays

In order to determine the MIC of these snake venoms, we tested them using the macro-dilution broth method as described elsewhere [13]. Briefly, after 5 h of the bacterial growth, the culture was diluted to obtain 1.0 × 10^5^ CFU mL^−1^. The snake venom was added in order to reach a final concentration from 0.5  *μ*g mL^−1^ to 1024  *μ*g mL^−1^ and incubated at 37°C for 24 h. MIC was defined as the lowest concentration of venom or antibiotic current on the market preventing visible bacterial growth compared to the positive growth control (medium plus bacteria without venom or antibiotic) that presented high turbidity, and to the negative growth controls (medium alone, medium plus venom or antibiotic and medium plus bacteria plus effective antibiotic) that presented no turbidity. All strains were tested at least in duplicate in four separate experiments and a reference antibiotic (vancomycin) was used as standard (MIC = 2 *μ*g mL^−1^).

### 2.3. Statistics

The statistical analyses were performed using Microcal Origin 4.0 (MA, USA). The SD and significance (*P*) were determined by using one-way ANOVA from the same graphic program. Results were considered as significant when *P* < .005.

## 3. Results

### 3.1. Sensibility Assays of Venoms

Our experimental data revealed that most of the venoms tested (*A*. *rhodostoma, B. atrox* and *B. jararaca*) exhibited an antibacterial profile against some of the Gram-positive bacteria (Table 1). The *L. muta* venom showed no antibacterial activity (Table 1) even when it was obtained from different Brazilian suppliers (Instituto Butantan, Ezequiel Dias and Vital Brazil) (data not shown).

*Agkistrodon rhodostoma* was not significantly effective against *S. aureus* and *E. coli* (Table 1). However, this venom was able to significantly inhibit *E. faecalis* and *S. epidermidis* growth (halo = 16 and 16 mm, resp.). *Bothrops atrox* venom also showed an antibiotic profile against *E. faecalis* and *S. epidermidis* (halo = 16 and 18 mm, resp.), different from *B. jararaca* venom, which acted only against *S. aureus* (halo = 16 mm) (Table 1). In addition, *Bothrops* venoms (*B. atrox* and *B. jararaca*) inhibited growth of *Staphylococcus* sp. (*S. epidermidis* and *S. aureus*, resp.).

### 3.2. MIC Assays of the Active Snake Venoms

**Figure 1:**
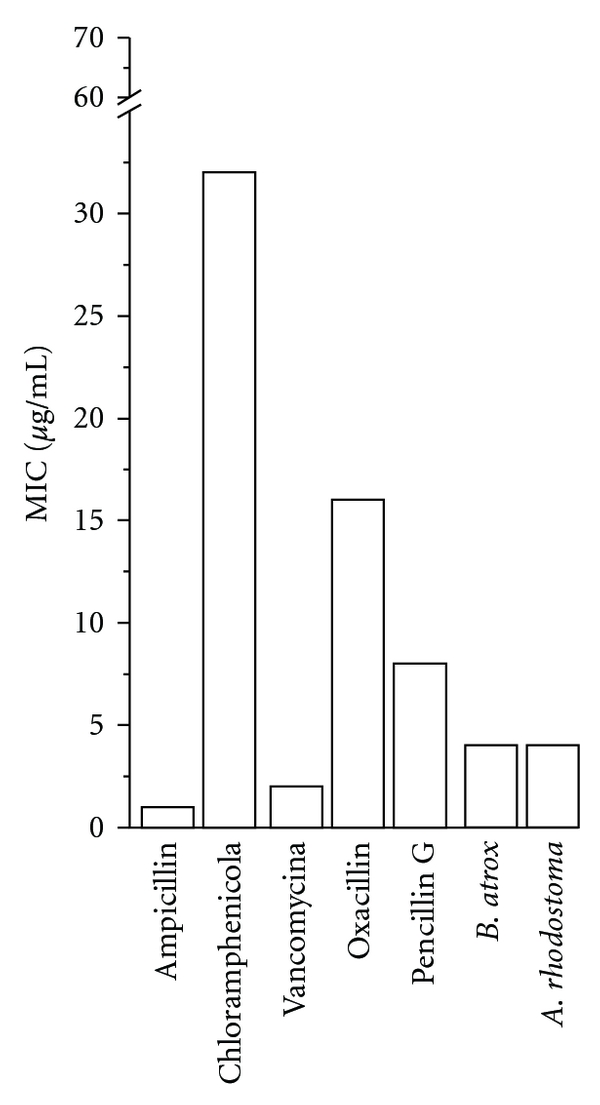
MIC of *Viperidae* venoms presenting halo >15 mm.

The MIC assays of the snake venoms that were active in the sensibility tests (*A. rhodostoma*, *B. atrox* and *B. jararaca*) revealed that their level of antibiotic activity was comparable (MIC = 4.5–13 *μ*g mL^−1^) (Table 2). *Agkistrodon rhodostoma and B. atrox* were also analogous to the antibiotics currently in use against *S. epidermidis*, such as ampicillin, chloramphenicol, vancomycin, oxacillin and penicillin G (MIC = 1–32 *μ*g mL^−1^) (Figure 1).

## 4. Discussion

Our experimental data revealed that most of the venoms tested (*A. rhodostoma, B. atrox* and *B. jararaca*) exhibited a promising antibacterial activity against some of the Gram-positive bacteria. Interestingly, despite of the presence of a known phospholipase A2 [14], the *L. muta* venom showed no antibacterial activity. Although literature described that different snake may present an individual pattern [5], *L. muta* different venom samples obtained from three different Brazilian suppliers (Governmental Institutes—Butantan, Ezequiel Dias and Vital Brazil) were not able to affect the bacterial strains (data not shown). This negative result reinforce the fact that the presence of enzymes in the snake venoms do not guarantee the antibiotic profile of these materials as the bacteria cell wall may avoid or affect the actions of these proteins against them.

Differently from *A. contortrix* venom [12], *A. rhodostoma* venom was able to significantly inhibit *E. faecalis* and *S. epidermidis* growth, which may suggest a specific mechanism or molecule of *A. rhodostoma* on affecting them.

*Bothrops atrox* venom also showed an antibiotic profile against *E. faecalis* and *S. epidermidis*, different from *B. jararaca* venom, which acted only against *S. aureus*. Recently, the literature described l-amino acid oxidases (L-MAO) isolated from *B. pirajai* [15] and *B. alternatus* venoms [16] able to inhibit *E. coli* growth. Our result pointed to the L-MAO isoforms presence in *Bothrops* sp. venoms as preserved components similar to C-type lectin-like proteins [17]. However, as the active profile of these venoms switched to different strains and not included *E. coli*, our result may also suggest that other antibacterial components may be present in these venoms resulted from species differentiation. In addition, *Bothrops* venoms (*B. atrox* and *B. jararaca*) acted against different *Staphylococcus* sp. (*S*. *epidermidis* and *S*. *aureus*, respectively) once again suggesting that different molecules and/or targets are involved in these biological activities. This hypothesis is reinforced by the presence of other different components found in snake venoms that sometimes are involved in a similar biological activity as RGD-peptides and some C-type lectin-like proteins from *B. jararaca* venom that are both platelet aggregation inhibitors [9].

Overall, our MIC assays revealed that the activity of the venoms tested was comparable (MIC = 4.5–13 *μ*g mL^−1^) among them. *Agkistrodon rhodostoma* and *B. atrox* were also analogous to the antibiotics currently in use against *S. epidermidis*, such as ampicillin, chloramphenicol, vancomycin, oxacillin and penicillin G (MIC = 1−32 *μ*g mL^−1^). Presently, *S. epidermidis* is an important nosocomial pathogen, drastically affecting immunocompromised patients and/or those with indwelling devices, such as joint prostheses, prosthetic heart valves and central venous catheters [18]. Therefore, these venoms' active profile against this strain is of interest for pursuing continuously an antibacterial molecule.

Proteomic technologies are improving and developing rapidly [19]. An important goal of proteomic studies of snake venoms is discovering molecules that may be used in treatment of diseases or as drugs prototypes. Nevertheless, these techniques may depend on the experimental data generated so far to identify some of these unknown proteins or peptides. Although snake venom peptides and proteins have a limited direct therapeutical use due to their antigenic and “digestible” structure, their usefulness as prototypes has clear potential [9, 20]. Our data suggested that *A. rhodostoma, B. atrox* and *B. jararaca* are feasible sources for searching antimicrobial prototypes and designing new antibiotics against drug-resistant clinical bacteria, and these data may act as a start for investing on these venoms proteomic study for prototypes searching.

## Figures and Tables

**Table 1:** Antibacterial effect of Viperidae venoms against Gram-positive and Gram-negative drug-resistant clinical bacteria.

Strain	Inhibition zone (mm)^a^			
	*A. rhodostoma*	*B. atrox*	*B. jararaca*	*L. muta*
*E. faecalis*	**16 ± 2**	**16 ± 2**	13 ± 1	1 ± 1
*S. epidermidis*	**16 ± 1**	**18 ± 2**	0	0
*S. aureus*	13 ± 2	12 ± 1	**16 ± 1**	2 ± 1
*E. coli*	11 ± 1	7 ± 1	5 ± 2	0
*S. morcencens*	8 ± 1	8 ± 2	7 ± 1	1 ± 1
*P. mirabilis*	7 ± 1	7 ± 1	7 ± 1	2 ± 1
*P. aeruginosa*	8 ± 2	8 ± 1	8 ± 1	1 ± 1
*E. calcoacetic*	9 ± 1	6 ± 2	6 ± 2	2 ± 1
*A. calcoacetic*	1 ± 2	2 ± 1	1 ± 1	1 ± 1
*K. pneumoniae*	9 ± 1	10 ± 1	10 ± 2	0

^a^The values represent a venom inhibition zone in millimeters, after l8 h incubation performed in triplicate assays (*P* < .005). Significant results were considered the halo >15 mm as vancomycin and ciprofloxacin presented halo = 15–17 and 23–25 mm, respectively.

**Table 2:** MIC of Viperidae venoms presenting halo >15 mm.

Strain^a^	MIC (*μ*g mL^−1^)		
	*A. rhodostoma*	*B. atrox*	*B. jararaca*
*E. faecalis*	4.5	4.5	—
*S. epidermidis* (A)	4.5	4.5	—
*S. epidermidis* (B)	4.5	4.5	—
*S. aureus* (A)	—	—	13
*S. aureus* (B)	—	—	13

^a^(A) and (B) on *S. aureus* and *S. epidermidis* refer to different patients strains.
